# Health Care Access and Use Among Adults With and Without Vision Impairment: Behavioral Risk Factor Surveillance System, 2018

**DOI:** 10.5888/pcd19.220066

**Published:** 2022-11-10

**Authors:** Qi Cheng, Catherine A. Okoro, Isabel Mendez, Elizabeth A. Lundeen, Jinan B. Saaddine, Renee Stein, Joseph Holbrook

**Affiliations:** 1 Division of Human Development and Disability, National Center on Birth Defects and Developmental Disabilities, Centers for Disease Control and Prevention, Atlanta, Georgia; 2 Division of Diabetes Translation, National Center for Chronic Disease Prevention and Health Promotion, Centers for Disease Control and Prevention, Atlanta, Georgia; 3 Community Guide Office, Office of the Associate Director for Policy and Strategy, Centers for Disease Control and Prevention, Atlanta, Georgia

## Abstract

**Introduction:**

Adults with vision impairment may have unique needs when accessing health care to maintain good health. Our study examined the relationship between vision status and access to and use of health care.

**Methods:**

We analyzed data on adults aged 18 years or older who participated in the 2018 Behavioral Risk Factor Surveillance System. Vision impairment was identified by a yes response to the question “Are you blind or do you have serious difficulty seeing, even when wearing glasses?” Survey questions assessed health care access over the past year (having health insurance coverage, a usual health care provider, or unmet health care needs because of cost) and use of health care during that period (routine checkup and dental visit). We estimated age-adjusted prevalence of our outcomes of interest and used bivariate analyses to compare estimates of the outcomes by vision impairment status.

**Results:**

The prevalence of self-reported vision impairment was 5.3%. Compared with adults without impaired vision, adults with vision impairment had a lower prevalence of having health insurance coverage (80.6% vs 87.6%), a usual health care provider (71.9% vs 75.7%), or a dental visit in the past year (52.9% vs 67.2%) and a higher prevalence of having an unmet health care need in the past year because of cost (29.2% vs 12.6%).

**Conclusion:**

Adults with vision impairment reported lower access to and use of health care than those without. Further research can better identify and understand barriers to care to improve access to and use of health care among this population.

**Summary**

**What is already known on this topic?**

Adults aged 40 years or older with impaired vision reported having more problems related to cost of health care and availability of insurance coverage according to pooled 2002–2004 data from the Medical Expenditure Panel Survey.

**What is added by this report?**

Adults aged 18 years or older with vision impairment reported lower access to and use of health care than those without, according to the 2018 Behavioral Risk Factor Surveillance System survey.

**What are the implications for public health practice?**

Further research can better identify and understand barriers to care to improve access to and use of health care among adults with vision impairment.

## Introduction

People with disabilities such as vision impairment often face challenges in receiving health care (1). In 2010, approximately 4 million US adults aged 40 years or older had impaired vision, and this number is projected to increase with the aging of the population and an increase in chronic conditions that lead to vision impairment (2,3). Vision impairment ranks among the top 10 disabilities in the US, and people with this disorder are more likely to have chronic conditions such as diabetes, heart disease, hypertension, injuries, depression, and premature death (4–11). They therefore have a particular need for increased access to general health care services to increase their use of those services.

High-quality and timely health care is essential to maintain good general health and prevent health disorders. Studies of people with impaired vision have focused on their access to and use of vision care; however, a better understanding is needed of how they access and use general health care services (12,13). A key objective of Healthy People 2030 is to increase the proportion of people with disabilities, including vision impairment, who have appropriate access to care and to eliminate disparities between people with and without disabilities (14). Gaps exist not only in our understanding of access to and use of general health care services but also of disparities in access and use between those with and without vision impairment (15,16). Our study aimed to describe the prevalence estimates of several measures of access to and use of general health care among US adults with and without impaired vision.

## Methods

The Behavioral Risk Factor Surveillance System (BRFSS) is an annual, state-based random–digit-dial landline and cellular telephone survey administered by states in collaboration with the Centers for Disease Control and Prevention (CDC). The survey is designed to collect information on health-related risk behaviors, chronic health conditions, and use of preventive services among noninstitutionalized adults aged 18 years or older residing in the US, the District of Columbia, and selected territories (ie, Guam and Puerto Rico). BRFSS uses 3 modules of questions: a core module that all jurisdictions collect, optional modules that focus on specific health issues that jurisdictions may choose to collect, and modules that jurisdictions can add according to their needs (11). The design, methodology, random sampling procedures, weighting strategies, and validity of measures for BRFSS have been previously published (17). We used the 2018 core module in our study, and the median survey response rate for all jurisdictions — 50 states, the District of Columbia, Guam, and Puerto Rico — was 49.9% (18). BRFSS data collection protocols are reviewed by CDC’s institutional review board (Protocol Number 2988) and the Office of Management and Budget (OMB No. 0920–1061, expiration date 3/31/2021). Secondary analyses are not subject to approval because data are de-identified. The overall sample consisted of 437,436 adults aged 18 years or older. We characterized respondents as having self-reported vision impairment if they answered yes to the question “Are you blind or do you have serious difficulty seeing, even when wearing glasses?” We excluded respondents who answered, “don’t know/not sure” or “refused” or had missing data in answers to this question (n = 11,134, 2.5%), yielding a sample of 426,302 adults.

Our outcome variables included several measures of health care access and use. Access was measured by using 3 variables: having health insurance coverage, having a usual health care provider, and having an unmet health care need. Having health insurance coverage was characterized as a yes response to the question “Do you have any kind of health insurance coverage, including health insurance, prepaid plans such as HMOs, or government plans such as Medicare or Indian Health Service?” Having a usual health care provider was indicated by a yes response to the question “Do you have one person you think of as your personal doctor or health care provider?” Having an unmet health care need because of cost in the past year was characterized as a yes response to the question, “Was there a time in the past 12 months when you needed to see a doctor but could not because of cost?” Health care use was measured by using 2 variables: having a routine checkup and having a dental visit. Receipt of a routine checkup within the past year was assessed by the question, “About how long has it been since you last visited a doctor for a routine checkup?” Having a dental visit in the past year was assessed by the question, “Including all types of dentists, such as orthodontists, oral surgeons, and all other dental specialists, as well as dental hygienists, how long has it been since you last visited a dentist or a dental clinic for any reason?” Responses to the 2 questions were dichotomized into in the past year or not in the past year.

Sociodemographic and geographic variables were age, sex, race or ethnicity, ratio of annual household income to the federal poverty level (FPL), and US region or territory of residence. We used the following categories to classify respondents: age (18–44, 45–64, or ≥65 y), sex, race or ethnicity (non-Hispanic White, non-Hispanic Black, Hispanic, and other races [American Indian/Alaska Native, Native Hawaiian or Pacific Islander, Asian, and multiracial]), and US region or territory (Northwest, Midwest, South, West, Guam, and Puerto Rico). FPL categories are based on the ratio of the respondent’s annual household income (given family size by numbers: 1–14 adults or children ≥0 y in the household) to the designated 2017 federal poverty threshold as defined by the US Census Bureau. This ratio was multiplied by 100 and expressed as a percentage, and federal poverty thresholds were then used to categorize respondents into 4 FPL categories: <100% of FPL, 100%–199% of FPL, ≥200% of FPL, or unknown (19). General health status was assessed by asking the question “Would you say that in general your health is?” They were coded into 3 categories: excellent/very good, good, or fair/poor. Health behavior variables of cigarette smoking and leisure-time physical activity were included. Cigarette smoking status was assessed by asking “Have you smoked at least 100 cigarettes in your entire life?” and “Do you now smoke cigarettes every day, some days, or not at all?” Respondents who reported smoking at least 100 cigarettes in their lifetime and who now smoke either every day or some days were coded as a “current smoker”; those who smoked at least 100 cigarettes in their lifetime and who now do not smoke at all were coded as “former smoker”; those who reported never having smoked 100 cigarettes in their lifetime were coded as “never smoker.” Leisure-time physical activity was assessed by asking “During the past month, other than your regular job, did you participate in any physical activities or exercises such as running, calisthenics, golf, gardening, or walking for exercise?” If respondents reported participating in any of these activities, they were coded as “engaging in leisure-time physical activity.” The metropolitan status classification was determined by applying the National Center for Health Statistics 2013 Urban−Rural Classification Scheme for Counties (20) to the BRFSS data using state and county FIPS (Federal Information Processing Standards) codes (https://www.nist.gov/standardsgov/compliance-faqs-federal-information-processing-standards-fips). Counties are considered metropolitan areas if they are classified as large central, large fringe, medium, or small metropolitan; counties are considered nonmetropolitan areas if they are classified as micropolitan or noncore. This classification was based on population density and metropolitan statistical areas (with an urbanized population ≥50,000 residents), micropolitan statistical areas (population cluster of between 10,000 and 49,999 residents), and noncore areas in the 50 states and District of Columbia (20). In addition to vision impairment, BRFSS included questions about 5 other disability types. We used these types as covariates and included hearing (“Are you deaf or do you have serious difficulty hearing?”), cognition (“Because of a physical, mental, or emotional condition, do you have serious difficulty concentrating, remembering, or making decisions?”), mobility (“Do you have serious difficulty walking or climbing stairs?”), self-care (“Do you have difficulty dressing or bathing?”), and independent living (“Because of a physical, mental, or emotional condition, do you have difficulty doing errands alone such as visiting a doctor’s office or shopping?”). Respondents who reported yes to any one of these questions were classified as having that disability. If a respondent answered yes to at least one disability other than vision impairment, they were classified as having any other disability; a respondent who answered no to all 5 other disabilities was classified as not having any other disability.

Sample sizes were computed by using unweighted numbers. Age-adjusted prevalence estimates and 95% CIs were calculated. Ninety-five percent CIs were calculated as the prevalence ±1.96 × standard error. Age standardization was performed by using the population distribution for the 3 age groups according to the 2000 US Census (18–44, 45–64, and ≥65 y), which remains the standard for the National Center for Health Statistics (21). SAS-callable SUDAAN, version 9.4 (Research Triangle Institute) was used to account for the multistage, complex sampling design.

## Results

Among surveyed adults, 23,545 reported having vision impairment, and the weighted prevalence of vision impairment was 5.3% (95% CI, 5.1%–5.4%). The characteristics of adults by self-reported vision impairment status are presented (Table 1).Respondents who were more likely to report having vision impairment were those aged 45 years and older (≥65 years, 31.5% vs 20.5%; 45–64 years, 39.8% vs 32.7%), women (56.2% vs 51.0% ), Hispanic respondents (25.3% vs 16.5%), non-Hispanic Black respondents (15.7% vs 11.5%), those living in lower-income households (<100% of FPL, 26.8% vs 12.1%; 100%–199% of FPL, 27.2% vs 18.8%), and those residing in the South US census region (43.3% vs 37.3%) and in the territories of Guam and Puerto Rico (3.9% vs 1.0%). Compared with respondents without vision impairment, those with impaired vision had a higher percentage of fair or poor general health (50.2% vs 16.8%), current cigarette smoking (24.7% vs 15.0%), and not engaging in leisure-time physical activity (41.1% vs 23.5%). Having 1 or more other disability was reported by a higher proportion of persons with vision impairment (68.2%) compared with those without vision impairment (23.2%). Compared with people without vision impairment, those with vision impairment reported a higher percentage of disability across all disability types (hearing: 22.6% vs 5.7%; cognition: 37.3% vs 10.0%; mobility: 44.9% vs 12.0%; self-care: 16.5% vs 3.1%; independent living: 29.4% vs 5.8%).

**Table 1:** Characteristics of Adults Aged ≥18 Years (N = 426,302) by Self-Reported Vision Impairment Status: US, Guam, and Puerto Rico, Behavioral Risk Factor Surveillance System, 2018

Characteristics	With vision impairment (n = 23,545) n (%) [95% CI]^a^	Without vision impairment (n = 402,757) n (%) [95% CI]^a^
**Age, y**		
18–44	3,777 (28.6) [27.3–30.0]	117,589 (46.8) [46.5–47.1]
45–64	9,505 (39.8) [38.4–41.3]	144,887 (32.7) [32.4–33.0]
≥65	10,263 (31.5) [30.2–32.9]	140,281 (20.5) [20.3–20.7]
**Sex**		
Male	9,684 (43.8) [42.3–45.3]	182,789 (49.0) [48.7–49.3]
Female	13,781 (56.2) [54.7–57.7]	219,089 (51.0) [50.7–51.3]
**Race or ethnicity**		
White, non-Hispanic	14,840 (51.3) [49.8–52.8]	303,356 (63.8) [63.5–64.2]
Black, non-Hispanic	2,838 (15.7) [14.6–16.8]	31,760 (11.5) [11.3–11.7]
Hispanic	3,134 (25.3) [23.8–26.9]	32,387 (16.5) [16.2–16.8]
Other^b^	2,182 (7.4) [6.8–8.8]	27,959 (8.2) [8.0–8.4]
**Percentage of federal poverty level^c^**		
<100	5,287 (26.8) [25.5–28.1]	36,470 (12.1) [11.9–12.3]
100–199	7,291 (27.2) [26.0–28.4]	77,863 (18.8) [18.5–19.0]
≥200	6,301 (24.9) [23.7–26.2]	222,397 (52.4) [52.1–52.7]
Unknown	4,666 (21.1) [19.8–22.6]	66,027 (16.7) [16.5–17.0]
**General health status^d^**		
Excellent/very good	4,900 (21.3) [20.1–22.6]	204,288 (50.9) [50.6–51.2]
Good	6,716 (28.4) [27.1–29.8]	127,853 (32.3) [32.0–32.6]
Fair/poor	11,802 (50.2) [48.7–51.7]	69,708 (16.8) [16.5–17.0]
**Cigarette smoking status^e^**		
Current smoker	5,625 (24.7) [23.5–25.9]	55,437 (15.0) [14.7–15.2]
Former smoker	7,078 (26.9) [25.7–28.1]	111,386 (24.0) [23.8–24.3]
Never smoker	10,444 (48.5) [46.9–50.0]	229,588 (61.0) [60.7–61.3]
**Leisure-time physical activity^f^**		
Yes	13,455 (58.9) [57.4–60.4]	305,216 (76.5) [76.2–76.8]
No	10,029 (41.1) [39.6–42.6]	97,005 (23.5) [23.2–23.8]
**Other disability type**		
Hearing	6,129 (22.6) [21.4–23.7]	32,361 (5.7) [5.6–5.9]
Cognition	8,103 (37.3) [35.9–38.8]	37,156 (10.0) [9.8–10.2]
Mobility	11,368 (44.9) [43.4–46.3]	60,057 (12.0) [11.8–12.2]
Self-care	4,005 (16.5) [15.5–17.4]	13,337 (3.1) [3.0–3.2]
Independent living	7,390 (29.4) [28.2–30.7]	24,195 (5.8) [5.7–6.0]
≥1 Other disability^g^	16,799 (68.2) [66.7–69.7]	105,138 (23.2) [22.9–23.5]
**Metropolitan status^h^**		
Metropolitan	14,423 (81.4) [80.5–82.3]	275,266 (85.1) [84.9– 85.3]
Nonmetropolitan	8,064 (18.6) [17.8– 19.5]	122,107 (14.9) [14.7–15.1]
**US Census region/territory**		
Northwest	4,164 (15.2) [14.3–16.2]	84,870 (17.5) [17.4–17.7]
Midwest	5,100 (17.5) [16.7–18.4]	103,931 (20.9) [20.7–21.0]
South	8,864 (43.3) [41.8–44.8]	123,414 (37.3) [37.1–37.5]
West	4,359 (20.2) [19.0–21.4]	85,221 (23.3) [23.2–23.5]
Guam and Puerto Rico	1,058 (3.9) [3.6–4.2]	5,321 (1.0) [0.9–1.0]

a Vision impairment was assessed by asking respondents, “Are you blind or do you have serious difficulty seeing, even when wearing glasses?” Answers were coded as yes or no. Data are weighted.

b American Indian or Alaska Native, Native Hawaiian or Pacific Islander, Asian, non-Hispanic, or multiracial.

c Federal poverty level (FPL) percentage was calculated as the ratio of the respondent’s annual household income to the appropriate simplified 2017 federal poverty threshold defined by the US Census Bureau (given family size: number of adults [from 1 to 14] in the household and number of children [≥0] in the household). This ratio is multiplied by 100 and expressed as a percentage, and FPL was then used to categorize respondents into 4 FPL groups: 1) <100% of FPL, 2) 100%–199% of FPL, 3) ≥200% of FPL, and 4) unknown.

d Determined by asking respondents, “Would you say that in general your health is — ?” Answers were categorized as excellent/very good, good, or fair/poor.

e Current smoker, smoked at least 100 cigarettes in their lifetime and smoked either every day or some days; former smoker, smoked at least 100 cigarettes in their lifetime and no longer smoked at all; never smoker, never smoked a total of 100 cigarettes in their lifetime.

f Categorized as participating in a leisure-time physical activity if they responded yes to the question “During the past month, other than your regular job, did you participate in any physical activities or exercises such as running, calisthenics, golf, gardening, or walking for exercise?”

g Categorized as have one or more other disabilities if response was yes to any 1 or more of 5 questions on hearing disability, cognition disability, mobility disability, self-care disability, or independent living disability.

h Categorized as metropolitan for respondents in counties classified as large central, large fringe, medium, or small metropolitan versus nonmetropolitan for respondents in counties classified as micropolitan or noncore, according to the National Center for Health Statistics 2013 Urban−Rural Classification Scheme (20).

Measures of health care access differed by vision impairment status (Table 2). Respondents with vision impairment had a lower prevalence of having health insurance coverage (80.6%; 95% CI, 78.9%–82.1% vs 87.6%; 95% CI, 87.3%–87.9%) and a usual health care provider (71.9%; 95% CI, 70.2%–73.5% vs 75.7%; 95% CI, 75.4%–76.0%), and a higher prevalence of reporting cost as a reason for unmet health care need (29.2%; 95% CI, 27.6%–31.0% vs 12.6%; 95% CI, 12.3%–12.8%), compared with those without vision impairment. The prevalence of these indicators for health care access also varied by vision impairment status within some sociodemographic and geographic subgroups. For example, among non-Hispanic White adults, those with vision impairment had a lower prevalence of having health insurance coverage and a usual health care provider compared with those without vision impairment; among Hispanic and non-Hispanic Black adults, we found no difference in the prevalence of having health insurance coverage or having a usual health care provider by vision impairment status. Respondents with vision impairment and 1 or more other disability types had a lower prevalence of having health insurance coverage. Compared with those without vision impairment, those with vision impairment reported a higher prevalence of cost as a reason for unmet health care needs in all subgroups.

**Table 2:** Weighted Age-Adjusted Prevalence^a^ Estimates for Health Care Access Measures^b^ Among Adults Aged ≥18 Years (N = 426,302), by Self-Reported Vision Impairment Status, Comorbid Disability Type, and Selected Characteristics: US, Guam, and Puerto Rico, Behavioral Risk Factor Surveillance System, 2018

Characteristics	Have health insurance coverage		Have a usual health care provider		Have unmet health care need because of cost	
	Vision impairment^c^	No vision impairment	Vision impairment^c^	No vision impairment	Vision impairment^c^	No vision impairment
**Total**	80.6 (78.9–82.1)	87.6 (87.3–87.9)	71.9 (70.2–73.5)	75.7 (75.4–76.0)	29.2 (27.6–31.0)	12.6 (12.3–12.8)
**Age, y**						
18–44	74.5 (71.6–77.2)	82.8 (82.4–83.2)	59.4 (56.5–62.3)	64.6 (64.1–65.1)	33.6 (30.8–36.5)	15.4 (15.1–15.8)
45–64	82.3 (80.5–84.0)	89.9 (89.5–90.3)	82.7 (81.1–84.2)	85.0 (84.6–85.4)	31.7 (29.6–33.9)	12.1 (11.8–12.5)
≥65	96.4 (95.3–97.2)	98.0 (97.8–98.2)	92.1 (90.1–93.8)	93.9 (93.6–94.2)	11.9 (10.2–13.9)	4.6 (4.3–4.9)
**Sex**						
Male	76.8 (74.2–79.3)	86.2 (85.8–86.6)	66.2 (63.5–68.7)	70.8 (70.4–71.2)	28.1 (25.6–30.8)	11.1 (10.8–11.4)
Female	83.8 (81.8–85.6)	88.8 (88.5–89.2)	76.9 (74.7–78.9)	80.6 (80.2–81.0)	30.2 (28.1–32.5)	14.1 (13.8–14.5)
**Race or ethnicity**						
White, non-Hispanic	84.2 (82.4–85.9)	91.5 (91.3–91.7)	75.7 (73.5–77.7)	78.5 (78.1–78.8)	28.4 (26.3–30.5)	10.9 (10.7–11.2)
Black, non-Hispanic	84.1 (80.4–87.2)	86.8 (86.1–87.5)	74.8 (70.7–78.5)	77.1 (76.2–77.9)	26.7 (23.0–30.7)	15.2 (14.4–16.0)
Hispanic	71.8 (68.1–75.3)	74.3 (73.4–75.3)	65.8 (62.0–69.4)	65.5 (64.5–66.5)	32.5 (28.9–36.3)	17.8 (17.0–18.6)
Other^d^	85.7 (81.7–89.0)	90.4 (89.5–91.3)	69.9 (64.7–74.5)	77.0 (75.9–78.1)	29.4 (24.1–35.3)	11.5 (10.7–12.3)
**Percentage of federal poverty level^e^**						
<100	74.1 (71.0–77.0)	75.7 (74.8–76.6)	70.3 (67.1–73.2)	68.7 (67.8–69.6)	35.6 (32.7–38.5)	22.7 (21.9–23.6)
100–199	81.7 (78.0–84.9)	81.6 (81.0–82.3)	70.0 (66.6–73.3)	71.0 (70.2–71.7)	33.5 (30.0–37.1)	19.1 (18.5–19.8)
≥200	88.4 (85.6–90.7)	93.7 (93.4–94.0)	76.9 (73.5–79.9)	80.0 (79.6–80.4)	20.0 (17.2–23.2)	7.9 (7.6–8.2)
Unknown	79.8 (76.1–83.1)	84.0 (83.2–84.7)	69.9 (65.9–73.7)	73.0 (72.2–73.8)	26.5 (22.5–30.8)	12.7 (12.1–13.3)
**General health status^f^**						
Excellent/very good	84.3 (81.4–86.9)	90.4 (90.0–90.7)	72.2 (69.2–75.0)	76.7 (76.3–77.1)	18.6 (16.1–21.5)	7.9 (7.6–8.2)
Good	79.0 (76.0–81.7)	85.4 (84.9–85.9)	70.8 (67.8–73.5)	74.3 (73.8–74.9)	27.2 (24.1–30.4)	14.1 (13.6–14.5)
Fair/poor	79.0 (76.3–81.6)	81.4 (80.6–82.3)	72.3 (69.5–74.9)	74.8 (74.0–75.7)	37.3 (34.6–40.2)	26.3 (25.4–27.1)
**Cigarette smoking status^g^**						
Current smoker	75.5 (72.6–78.3)	81.9 (81.2–82.6)	64.5 (61.5–67.3)	68.4 (67.6–69.2)	37.7 (34.8–40.7)	20.2 (19.5–20.9)
Former smoker	82.4 (77.6–86.4)	89.1 (88.4–89.6)	75.3 (70.7–79.4)	77.5 (76.8–78.1)	27.1 (23.5–31.1)	12.7 (12.2–13.2)
Never smoker	82.5 (80.2–84.7)	88.6 (88.2–88.9)	74.6 (72.3–76.8)	77.1 (76.7–77.4)	26.0 (23.6–28.5)	10.7 (10.4–11.0)
**Leisure-time physical activity^h^**						
Yes	80.8 (78.7–82.7)	89.2 (88.9–89.5)	72.7 (70.7–74.6)	76.8 (76.4–77.1)	28.6 (26.6–30.7)	11.5 (11.2–11.7
No	80.3 (77.4–82.9)	81.1 (80.4–81.8)	69.4 (66.2–72.3)	71.4 (70.6–72.1)	30.2 (27.5–33.1)	16.4 (15.8–17.1)
**Other disability type**						
Hearing	79.7 (74.5–84.0)	84.7 (83.1–86.2)	72.5 (67.4–77.0)	73.5 (71.4–75.4)	37.5 (32.7–42.5)	22.0 (20.3–23.9)
Cognition	79.6 (76.9–82.1)	84.9 (84.1–85.7)	74.1 (71.5–76.6)	76.9 (76.0–77.8)	38.3 (35.7–41.1)	27.6 (26.7–28.6)
Mobility	83.4 (80.4–86.0)	86.3 (85.2–87.4)	80.1 (76.8–83.0)	82.9 (81.8–84.0)	38.9 (35.6–42.2)	25.8 (24.6–27.1)
Self–care	83.1 (78.0–87.1)	85.5 (82.5–88.1)	79.9 (75.1–84.0)	83.9 (81.9–85.7)	43.3 (38.4–48.3)	28.8 (26.5–31.2)
Independent living	85.4 (82.9–87.7)	86.7 (85.5–87.7)	79.0 (75.9–81.8)	79.5 (78.2–80.8)	36.6 (33.5–39.9)	29.0 (27.9–30.7)
≥1 other disability type^i^	81.3 (79.2–83.3)	85.2 (84.5–85.8)	74.8 (72.7–76.9)	77.4 (76.7–78.1)	34.8 (32.6–37.1)	24.6 (23.9–25.3)
**Metropolitan status^j^**						
Metropolitan	80.4 (78.5–82.2)	87.7 (87.4–88.0)	71.3 (69.3–73.1)	75.6 (75.2–75.9)	29.3 (27.4–31.4)	12.5 (12.3–12.8)
Nonmetropolitan	78.9 (75.9–81.7)	86.3 (85.7–87.0)	73.3 (70.1–76.4)	76.1 (75.4–76.8)	30.9 (27.9–34.1)	13.0 (12.5–13.5)
**US Census region/territory**						
Northwest	85.2 (82.0–87.9)	90.6 (90.1–91.1)	78.6 (75.1–81.7)	81.1 (80.5–81.7)	21.7 (18.6–25.2)	10.3 (9.9–10.8)
Midwest	84.8 (81.7–87.5)	89.9 (89.6–90.3)	76.2 (73.0–79.2)	78.8 (78.3–79.3)	26.4 (23.6–29.4)	11.0 (10.6–11.3)
South	76.2 (73.3–79.0)	84.0 (83.4–84.5)	69.3 (66.3–72.1)	73.0 (72.4–73.6)	33.7 (30.8–36.7)	15.1 (14.7–15.6)
West	81.3 (78.1–84.1)	88.5 (88.1–89.0)	68.0 (64.4–71.4)	73.0 (72.3–73.6)	28.9 (25.5–32.5)	11.7 (11.3–12.2)
Guam and Puerto Rico	90.0 (86.0–92.9)	90.7 (89.4–91.9)	81.7 (77.2–85.5)	80.4 (78.7–81.9)	22.6 (18.5–27.3)	12.5 (11.2–13.9)

a Age adjustment for prevalence standardized based on the population breakdown for the following age groups according to the 2000 US Census: 18–44, 45–64, and ≥65 years (http://www.cdc.gov/nchs/data/statnt/statnt20.pdf). Values are weighted percentage (95% CI).

b Health care access measures include 3 measures of “Had health insurance coverage,” “Had usual health care provider,” and “Had unmet health care need because of cost in the past year.” Responses for each health care access outcome were dichotomized into yes or no respectively.

c Vision impairment was assessed by asking respondents, “Are you blind or do you have serious difficulty seeing, even when wearing glasses?” Answers were coded as yes or no.

d American Indian or Alaska Native, Native Hawaiian or Pacific Islander, Asian, non-Hispanic, or multiracial.

e Federal poverty level (FPL) percentage was calculated as the ratio of the respondent’s annual household income to the appropriate simplified 2017 federal poverty threshold defined by the US Census Bureau (given family size: number of adults [from 1 to 14] in the household and number of children [≥0] in the household). This ratio is multiplied by 100 and expressed as a percentage, and FPL was then used to categorize respondents into 4 FPL groups: 1) <100% of FPL, 2) 100%–199% of FPL, 3) ≥200% of FPL, and 4) unknown.

f Assessed by asking “Would you say that in general your health is excellent/very good, good, or fair/poor?”

g Current smoker, smoked at least 100 cigarettes in their lifetime and smoked either every day or some days; former smoker, smoked at least 100 cigarettes in their lifetime and no longer smoked at all; never smoker, never smoked a total of 100 cigarettes in their lifetime.

h Respondents were categorized as participating in a leisure-time physical activity if they responded yes to a question of “During the past month, other than your regular job, did you participate in any physical activities or exercises such as running, calisthenics, golf, gardening, or walking for exercise?”

i Adults were categorized as have one or more other disabilities if they responded yes to any 1 or more of 5 questions on hearing disability, cognition disability, mobility disability, self-care disability, or independent living disability.

j Metropolitan status was categorized as a Metropolitan area for respondents in counties classified as large central, large fringe, medium, or small metropolitan versus Nonmetropolitan area for respondents in counties classified as micropolitan or noncore, according to the National Center for Health Statistics 2013 Urban−Rural Classification Scheme (20).

Prevalence of receiving health and dental care in the past year differed by vision impairment status (Table 3). We found no difference in the receipt of a routine health checkup in the past year between respondents with or without vision impairment (75.6%; 95% CI, 73.9%–77.2% vs 75.2%; 95% CI, 74.9%–75.5%). Among those with vision impairment, just over half (52.9%; 95% CI, 51.2%–54.7%) reported that they had a dental visit in the past year, which was lower than those without vision impairment (67.2%; 95% CI, 66.9%–67.6%). The prevalence of having had a dental visit in the past year was lower among those with vision impairment, compared with those without vision impairment; this was the case in all subgroup comparisons with the exception of Hispanic respondents and adults with a disability affecting self-care or independent living.

**Table 3:** Weighted Age-Adjusted Prevalence^a^ Estimates for Use of Health Care Measures^b^ Among Adults Aged ≥18 years, by Self-reported Vision Impairment Status, Comorbid Disability Type, and Selected Characteristics: US, Guam, and Puerto Rico, Behavioral Risk Factor Surveillance System, 2018

Characteristic	Had routine checkup within the past year, % (95% CI)		Had a dental visit within the past year, % (95% CI)	
	Vision impairment^c^	No vision impairment	Vision impairment^c^	No vision impairment
**Total**	75.6 (73.9–77.2)	75.2 (74.9–75.5)	52.9 (51.2–54.7)	67.2 (66.9–67.6)
**Age, y**				
18–44	66.2 (63.2–69.0)	67.4 (66.9–67.9)	56.7 (53.8–59.5)	65.1 (64.6–65.6)
45–64	82.1 (80.4–83.7)	79.9 (79.4–80.3)	48.9 (46.6–51.3)	69.6 (69.1–70.1)
≥65	93.2 (92.1–94.1)	91.7 (91.3–92.0)	49.0 (46.4–51.7)	69.4 (68.8–70.0)
**Sex**				
Male	70.6 (68.0–73.2)	71.0 (70.6–71.5)	50.4 (47.8–53.1)	64.3 (63.8–64.8)
Female	79.7 (77.7–81.6)	79.5 (79.1–79.9)	55.4 (53.1–57.6)	70.0 (69.6–70.5)
**Race or ethnicity**				
White, non-Hispanic	74.7 (72.6–76.7)	74.2 (73.8–74.5)	50.4 (48.2–52.7)	70.0 (69.6–70.4)
Black, non-Hispanic	83.5 (79.5–86.8)	83.8 (83.0–84.6)	51.7 (47.6–55.8)	61.2 (60.2–62.2)
Hispanic	72.6 (69.1–75.9)	72.8 (71.8–73.7)	56.7 (52.9–60.4)	59.8 (58.6–60.9)
Other^d^	82.0 (72.7–88.6)	74.6 (71.5–77.4)	54.0 (47.8–60.0)	67.7 (66.3–69.0)
**Percentage of federal poverty level^e^**				
<100	75.7 (73.0–78.2)	74.0 (73.1–74.9)	43.6 (40.5–46.8)	49.7 (48.6–50.7)
100–199	73.2 (69.5–76.5)	72.7 (71.9–73.4)	47.5 (44.1–51.0)	54.8 (54.0–55.6)
≥200	76.1 (72.7–79.1)	76.4 (76.0–76.8)	67.9 (64.7–71.0)	75.6 (75.2–76.0)
Unknown	77.1 (72.8–80.8)	75.9 (75.1–76.7)	54.7 (50.4–59.0)	66.0 (65.1–66.9)
**General health status^f^**				
Excellent/very good	74.4 (71.4–77.3)	74.3 (73.9–74.7)	64.4 (61.3–67.4)	74.4 (74.0–74.8)
Good	72.2 (69.1–75.2)	75.3 (74.8–75.9)	55.6 (52.6–58.6)	63.7 (63.1–64.3)
Fair/poor	77.1 (74.5–79.6)	77.0 (76.2–77.9)	45.3 (42.4–48.2)	52.1 (51.1–53.0)
**Cigarette smoking status^g^**				
Current smoker	71.4 (68.7–73.9)	67.9 (67.1–68.7)	40.4 (37.4–43.5)	50.0 (49.1–50.8)
Former smoker	77.0 (72.7–80.9)	75.6 (74.9–76.3)	52.5 (48.1–56.9)	66.0 (65.2–66.7)
Never smoker	76.6 (74.1–78.9)	76.8 (76.4–77.1)	59.6 (57.2–62.0)	71.8 (71.4–72.2)
**Leisure-time physical activity^h^**				
Yes	74.9 (72.8–76.8)	75.4 (75.1–75.7)	56.7 (54.6–58.7)	70.6 (70.3–71.0)
No	76.0 (73.0–78.7)	74.2 (73.5–74.9)	47.7 (44.5–50.9)	56.3 (55.6–57.1)
**Other disability type**				
Hearing	72.9 (67.7–77.6)	73.8 (71.6–75.8)	46.5 (41.4–51.7)	58.6 (56.5–60.7)
Cognition	77.3 (74.8–79.6)	77.3 (76.4–78.1)	47.5 (44.8–50.3)	54.4 (53.3–55.4)
Mobility	83.2 (80.6–85.6)	83.4 (82.3–84.4)	46.0 (42.7–49.4)	52.8 (51.4–54.2)
Self-care	80.8 (75.9–84.9)	83.4 (81.3–85.3)	49.0 (44.0–54.1)	50.9 (48.3–53.6)
Independent living	81.0 (78.1–83.6)	79.4 (78.1–80.6)	46.6 (43.4–49.9)	50.0 (48.5–51.5)
≥1 other disability type^i^	78.3 (76.3–80.2)	77.7 (77.0–78.4)	49.2 (47.0–51.5)	55.8 (55.0–56.5)
**Metropolitan status^j^**				
Metropolitan	75.6 (73.6–77.4)	75.5 (75.1–75.8)	54.2 (52.2–56.2)	68.2 (67.9–68.6)
Nonmetropolitan	74.4 (71.1–77.4)	73.7 (73.0–74.3)	44.4 (41.2–47.7)	61.1 (60.4–61.8)
**US Census region/territory**				
Northwest	81.3 (78.0–84.1)	78.7 (78.1–79.4)	58.5 (54.8–62.1)	71.4 (70.7–72.1)
Midwest	76.6 (73.6–79.4)	75.6 (75.1–76.1)	56.4 (53.1–59.7)	68.9 (68.4–69.5)
South	75.6 (72.6–78.3)	76.3 (75.7–76.9)	49.7 (46.7–52.7)	63.8 (63.2–64.4)
West	70.1 (66.6–73.4)	70.5 (69.9–71.2)	51.0 (47.2–54.7)	67.7 (67.1–68.4)
Guam and Puerto Rico	80.1 (75.5–84.0)	79.0 (77.2–80.6)	68.0 (64.0–71.9)	71.2 (69.4–72.9)

a Age adjustment for prevalence was standardized based on the population breakdown for the following age groups according to the 2000 US Census: 18–44, 45–64, and ≥65 years (http://www.cdc.gov/nchs/data/statnt/statnt20.pdf).

b Use of health care included 2 measures, having a routine checkup within the past year and having a dental visit within the past year. Responses to each were dichotomized as yes or no.

c Vision impairment was assessed by asking respondents, “Are you blind or do you have serious difficulty seeing, even when wearing glasses?” Answers were coded as yes or no.

d American Indian or Alaska Native, Native Hawaiian or Pacific Islander, Asian, non-Hispanic, and multiracial.

e Federal poverty level (FPL) percentage was calculated as the ratio of the respondent’s annual household income to the appropriate simplified 2017 federal poverty threshold defined by the US Census Bureau (given family size: number of adults [from 1 to 14] in the household and number of children [≥0] in the household). This ratio is multiplied by 100 and expressed as a percentage, and FPL was then used to categorize respondents into 4 FPL groups: 1) <100% of FPL, 2) 100%–199% of FPL, 3) ≥200% of FPL, and 4) unknown.

f Assessed by asking “Would you say that in general your health is excellent/very good, good, or fair/poor?”

g Current smoker, smoked at least 100 cigarettes in their lifetime and smoked either every day or some days; former smoker, smoked at least 100 cigarettes in their lifetime and no longer smoked at all; never smoker, never smoked a total of 100 cigarettes in their lifetime.

h Categorized as participating in a leisure-time physical activity if they responded yes to the question “During the past month, other than your regular job, did you participate in any physical activities or exercises such as running, calisthenics, golf, gardening, or walking for exercise?”

i Categorized as having one or more other disabilities if response was yes to any 1 or more of 5 questions on hearing disability, cognition disability, mobility disability, self-care disability, or independent living disability.

j Categorized as metropolitan for respondents in counties classified as large central, large fringe, medium, or small metropolitan versus nonmetropolitan for respondents in counties classified as micropolitan or noncore, according to the National Center for Health Statistics 2013 Urban−Rural Classification Scheme (20).

## Discussion

Our results indicate that BRFSS respondents with impaired vision had a lower prevalence of having health insurance coverage, a usual health care provider, or a dental visit in the past year and a higher prevalence of having unmet health care needs because of cost. Also, we found little to no difference in the prevalence of having had a routine checkup in the past year between adults with and without vision impairment. These findings suggest that people with impaired vision face greater barriers to health care access and use than those without. Results of our analysis may contribute to a further understanding of these barriers. A previous study on this topic used 2002–2004 Medical Expenditure Panel Survey data (22,23), and as in our study, found that people with vision impairment had more access problems related to cost of care than those without. Importantly, that study found adults with vision impairment were more likely to have insurance coverage whereas we found that adults aged younger than 65 years with vision impairment were less likely to have insurance coverage. Although our study found that a similar percentage of adults aged 65 years or older reported having insurance coverage, we could not rule out the possibility that Medicare coverage would contribute to this observation. Thus, further investigation is warranted on this topic. Sufficient access to and use of health care services, including preventive care, is especially important for people with vision impairment to maintain health, because they are more likely to have other health disorders or comorbidities (5,24–26). Previous study findings indicated that older US adults (≥50 y) with vision impairment may be less likely to use cancer-related preventive services than those without (27). That study found the prevalence of self-reported poor health status was 3 times higher among people with vision impairment compared with those without vision impairment. Although that cross-sectional study did not allow for examination of causality, this disparity may be partially due to people with vision impairment receiving inadequate medical treatment and preventive care.

A recent study that focused on US adults aged 40 years or older found that vision-impaired people received fewer regular dental preventive care and treatment services (28). Poor dental care can have a negative impact on self-esteem, quality of life, nutrition, communication, and general health. However, that study did not assess whether other factors, such as comorbidities or low-income status, accounted for some of the association between vision impairment and less use of dental care.

Respondents with vision impairment were more than twice as likely to report unmet health care needs in the past year because of cost than those without impaired vision. In addition, a household income below the federal poverty threshold was reported more often by respondents with vision impairment than those without. Taken together, these data suggest that cost may pose a substantial health care barrier for people with vision impairment. Spencer and colleagues reported that transportation issues and refusal of services by health care providers were primary barriers among people aged 40 years or older with vision impairment (22). Challenges associated with transportation are likely to affect people with vision impairment more than others, and people with disabilities have limited access to health care facilities because of a lack of assistive technology (29). The use of assistive technology, such as application of the sighted guide technique and screen magnifiers for low-vision computer users, could help reduce this disparity. Additionally, social support is an important consideration in obtaining access to health care among people with vision impairment (30). In all, the factors associated with barriers to access and use of health and dental care services among people with impaired vision are complex and multidimensional and can exist at the individual, population, and societal level. Our study considered broad associations, whereas future studies could examine how specific factors interact with characteristics of vision-impaired people. Understanding how such factors are associated with barriers to access and use of health and dental care among people with vision impairment requires further study.

Our study had limitations that should be considered when interpreting its findings. Health care access and use among people with impaired vision may be affected by many factors, and causality cannot be inferred from our analysis. The question defining vision disability asked in the BRFSS survey may capture only serious forms of vision impairment; therefore, any associations found in our study may not represent associations among people with mild or moderate vision impairment. Because BRFSS data are cross-sectional, causality among sociodemographic characteristics, health care access and use, and vision impairment cannot be inferred. Similarly, longitudinal information (eg, length of time the person had impaired vision, cause of vision impairment) was not included in our analyses. Lastly, because BRFSS data are self-reported, they may be subject to recall error.

In conclusion, by using a nationally drawn sample of US noninstitutionalized adults, we found that people with vision impairment had lower access to and use of health care services than people without vision impairment, specifically a lower prevalence of having health insurance coverage, a usual health care provider, and receiving dental care, and a higher prevalence of having unmet health care needs because of cost within the past year. Further work would better elucidate barriers to access and use of health care services to remove these barriers and improve the health of people with impaired vision.
